# *M. tuberculosis* Beijing Genotype Clusters: Impact on Tuberculosis Drug Resistance in Kazakhstan

**DOI:** 10.3390/microorganisms14092121

**Published:** 2026-09-21

**Authors:** Ainur Akhmetova, Zhannur Abilova, Dauren Yerezhepov, Lyailya Chingissova, Ainur Akilzhanova, Ulan Kozhamkulov

**Affiliations:** 1 Laboratory of Genomic and Personalized Medicine, Center for Life Sciences, National Laboratory Astana, Nazarbayev University, Astana 010000, Kazakhstan; 2 National Scientific Center of Phthisiopulmonology of the Republic of Kazakhstan, Almaty 050000, Kazakhstan

**Keywords:** tuberculosis, Beijing genotype, Central Asian/Russian cluster 94-32, drug resistance, clinical forms

## Abstract

The Beijing genotype is the main component of the *M. tuberculosis* population in Kazakhstan. In this study, we investigated the association of drug resistance (including MDR) and clinical forms of tuberculosis with the Beijing genotype and its dominant clusters, and compared obtained results among new, recurrent and all TB cases. In total, 759 *M. tuberculosis* isolates were collected from new (*n* = 540) and recurrent TB cases (*n* = 219). Drug susceptibility testing was performed on the BACTEC-MGIT 960 system; 24 MIRU-VNTR analysis was carried out to identify *M. tuberculosis* genotypes. The Beijing genotype was detected in 65.2% (495/759) of all TB cases and was associated with recurrent TB compared with new cases (78% vs. 60%, *p* < 0.0001). Among both new and recurrent TB cases, the Beijing genotype was associated with drug resistance (*p* < 0.0001 and *p* < 0.0001), including MDR (*p* < 0.0001 and *p* = 0.0118). Central Asian/Russian cluster 94-32 was the most widespread Beijing cluster in Kazakhstan (52.5%; 260/495). Association of cluster 94-32 with drug-resistant TB in general was confirmed across all cases (*p* = 0.010) and recurrent TB cases (*p* = 0.0191; OR = 4.8958; 95%CI: 1.2965–18.4883), while individuals with new TB cases were more likely to be diagnosed with MDR-TB specifically (*p* = 0.021). Among clinical forms of TB, a significant association was detected between fibrous–cavernous tuberculosis and drug resistance (*p* = 0.0019; OR = 3.3801; 95%CI: 1.5692–7.2807), including MDR (*p* = 0.0054; OR = 3.2453; 95%CI: 1.4167–7.4345), among all TB cases. TB patients with Beijing cluster 94-15 mostly had drug-susceptible TB among all TB cases and recurrent cases (*p* < 0.05). Drug-susceptible tuberculosis was observed among patients with focal TB among new cases (*p* = 0.0254; OR = 0.1723; 95%CI: 0.0369–0.8050). Tuberculous pleurisy was more commonly diagnosed in individuals with non-Beijing genotypes among all TB cases (*p* = 0.0430; OR = 0.3643; 95%CI: 0.1371–0.9686). The *M. tuberculosis* Beijing genotype, particularly the Central Asian cluster 94-32, plays a significant role in the distribution of drug-resistant TB among all TB cases and recurrent cases, including MDR-TB among new TB cases in Kazakhstan. On the contrary, the Beijing cluster 94-15 may contribute to the spread of drug-susceptible TB among all TB cases and recurrent TB cases.

## 1. Introduction

Understanding the clinical importance of *Mycobacterium tuberculosis* population diversity remains one of the main problems in molecular epidemiology and personalized medicine of tuberculosis. Strains of different *M. tuberculosis* lineages showed differences in the acquisition of resistance to anti-TB drugs, rates of in vitro growth and virulence in laboratory animals. *M. tuberculosis complex* (MTBC) includes nine genetic lineages. Lineages L1–L4 and L7–L9 belong to *M. tuberculosis;* lineages L5 and L6 relate to *M. africanum* [1,2]. Genotypes of lineages L2 and L4 were associated with high transmissibility and severe disease progression in various studies. Not only the specific *M. tuberculosis* strain characteristics but also other factors such as early diagnostics, treatment effectiveness, HIV coinfection, host genetics and social factors affect the outcome of the disease [3,4].

The Beijing genotype is the main representative of lineage L2 (East Asian lineage), which is a widespread and well-studied lineage [5]. In the past 30 years, Beijing isolates have caused tuberculosis outbreaks in the United States [6] and Europe [7,8]. The Beijing genotype is mainly associated with multidrug resistance (MDR) [9]. However, there are some regions where the Beijing genotype has not been linked to drug resistance [10], indicating that various clusters of the Beijing genotype are distributed in different geographic locations.

The use of molecular phylogeny markers and high-resolution genotyping approaches enabled the scientific community to investigate the population structure of the Beijing genotype more deeply and to identify clusters with particular characteristics of clinical and epidemiological significance. The W-Beijing strain was found during the MDR-TB outbreak in New York (USA) in the mid-1990s [6]. The highly transmissible Gran Canaria GC1237 strain was detected in an African refugee from Liberia in the Spanish island of Gran Canaria in the late 1990s and was a source of rapid transmission of tuberculosis in the community [11]. Modern Beijing genotype clusters, Central Asian/Russian cluster 94-32 and Russian cluster 100-32, are mostly circulating in the former USSR countries, and are associated with MDR [12,13,14]. The ancient Beijing clusters, which were identified in the territory of the Russian Federation, cluster 1071-32 (Siberia) and cluster 14717-15 (Far East), had an association with MDR/XDR-TB [15,16]. Also, isolates of cluster 14717-15 revealed associations with hypervirulence and high lethality in mouse models [16].

In our previous study, we showed that the Beijing genotype is the most distributed *M. tuberculosis* genotype in Kazakhstan [12]. Among clinical isolates of *M. tuberculosis* from new TB cases, the Beijing genotype was identified in 60% of cases. MDR-TB was strongly associated with the Beijing genotype, and among Beijing clusters, the Central Asian/Russian cluster 94-32 was associated with MDR (*p* < 0.05) [12]. The association of the Beijing genotype and its main clusters with the most widely spread mutations, Ser531Leu and Ser315Thr, in the *rpoB* and *katG* genes, responsible for rifampicin and isoniazid resistance, respectively, was evaluated [12].

Collecting *M. tuberculosis* isolates only from new TB cases limits sampling for assessing the prevalence of drug resistance in the general population. Therefore, to identify the Beijing genotype and its features in the Kazakhstani *M. tuberculosis* population, it is necessary to study this genotype among TB patients with new and recurrent cases, including all currently available samples (resistant and susceptible).

Earlier whole-genome sequencing results were reported for Beijing isolates from Kazakhstan with different drug resistance profiles (MDR, pre-XDR, and XDR) [17,18,19]. However, no studies in Kazakhstan have assessed the relationship between the Beijing genotype and its dominant clusters and clinical forms of tuberculosis.

This study aimed to evaluate the association between drug resistance (including MDR), clinical forms of tuberculosis, and the Beijing genotype and its dominant clusters, and to compare the results among new, recurrent, and all TB cases (new + recurrent TB cases).

## 2. Materials and Methods

### 2.1. M. tuberculosis Samples and Drug Susceptibility Testing

In total, 759 clinical isolates of *M. tuberculosis* were gathered in 2018–2023 from 759 patients with pulmonary tuberculosis, including new (*n* = 540) and recurrent TB cases (*n* = 219) from all regions of Kazakhstan: North Kazakhstan (*n* = 179), South Kazakhstan (*n* = 405), East Kazakhstan (*n* = 85) and West Kazakhstan (*n* = 90). The sample collection included all clinical isolates (susceptible and resistant) obtained from patients who visited regional TB dispensaries during the study and were grown on solid nutrient media. Exclusion criteria of *M. tuberculosis* isolates in the study were insufficient quantity of *M. tuberculosis* lysates, duplicates of the samples, and a lack of Drug Susceptibility Testing (DST) results.

Identification and isolation of *M. tuberculosis* were performed at the National Reference Laboratory of the National Scientific Center of Phthisiopulmonology in Almaty, Kazakhstan. Epidemiological and clinical data, such as age, gender, clinical forms of TB, and DST results of the *M. tuberculosis* isolates, were obtained for all TB patients. DST for four first-line anti-TB drugs was done using the BACTEC-MGIT 960 system (BD Diagnostic System, Franklin Lakes, NJ, USA) using the following critical drug concentrations: rifampicin—0.5 µg/mL; isoniazid—0.1 µg/mL; ethambutol—5 µg/mL; and streptomycin—1 µg/mL.

### 2.2. Genotyping of M. tuberculosis Clinical Isolates

DNA isolation from clinical isolates of *M. tuberculosis* was carried out according to a previously described protocol [20].

We performed 24 MIRU-VNTR genotyping of 759 *M. tuberculosis* samples as described earlier [21]. A web resource, www.miru-vntrplus.org, (accessed on 1 February 2024) was used to compare the obtained MIRU-VNTR digital profiles of the isolates and to identify *M. tuberculosis* genotypes.

RD typing was also performed to confirm Beijing isolates. RD analysis was performed on RD105 loci for Beijing genotype *M. tuberculosis* isolates (*n* = 495), as shown before [12,22,23].

### 2.3. Statistical Analysis

To identify any significant differences between two categorical variables, Chi-square (χ2) and Fisher’s Exact Tests were conducted using IBM SPSS Statistics 24.0 software [24]. If the expected cell frequency was below 10, then χ2 with Yates’ correction was accessed [25]. χ2 with Yates’ correction and *p*-values were calculated with 95% confidence intervals (CI) for the mean using MedCalc online (version 22.009, Ostend, Belgium) [26].

Obtained results were considered statistically significant if the *p*-value was equal to or less than 0.05. If the *p*-value was greater than 0.05, then the null hypothesis was not rejected.

In our study, statistical analysis was performed for three groups of samples for determination of the significance of obtained results and comparison of groups: all cases (*n* = 759) and samples isolated from new (*n* = 540) and recurrent (*n* = 219) TB cases.

## 3. Results

### 3.1. Characteristics of TB Patients

Among the 759 TB patients included in this research, 64.7% (491/759) were men and 35.3% (268/759) were women. The mean age of the study participants was 37.4 years.

In our study, among the 759 TB patients, infiltrative pulmonary tuberculosis was diagnosed in 86.3% of cases. Fibrous–cavernous tuberculosis and tuberculosis pleurisy were diagnosed in 7% and 2.23% of patients, respectively. Other clinical forms of TB were determined in less than 2% of cases: disseminated tuberculosis—1.8%; focal pulmonary tuberculosis—1.44%; cirrhotic tuberculosis—0.13%; miliary tuberculosis—0.4%; primary tuberculosis complex—0.4%; and caseous pneumonia—0.3% (Table 1).

Clinical characteristics of the patients among new and recurrent cases separately and among all cases are shown in Table 1.

### 3.2. Drug Susceptibility Testing (DST) Results

DST results showed that among all cases, 64% (486/759) of the clinical isolates were resistant and 36% (273/759) were susceptible to the first-line anti-TB drugs used in TB treatment. Drug-resistant isolates were more prevalent among recurrent TB cases (84.9%, 186/219) than among new cases (55.6%, 300/540) (*p* < 0.0001).

Distributions of the monoresistant, polyresistant and MDR isolates and their various resistance combinations among new and recurrent cases and among all cases are presented in Table 2.

Among drug-resistant TB cases (*n* = 486), 64.6% of the isolates had multidrug resistance (MDR), the most dangerous form of drug resistance (DR) that is associated with resistance to the most effective first-line anti-TB drugs, rifampicin and isoniazid. Four drug resistance combinations were observed among MDR samples, and 48.3% of the isolates had resistance to all tested first-line anti-TB drugs (Table 2). MDR was more commonly detected among recurrent cases (84.4%) than among new cases (52.3%) (*p* < 0.0001).

Among drug-resistant *M. tuberculosis* isolates, 22.8% were polyresistant (resistant to two or more drugs, excluding the RMP + INH combination) and 12.6% were monoresistant (resistant to one drug). Almost half of the monoresistant isolates (6%; *n* = 29) were resistant to streptomycin (Table 2).

### 3.3. Genotyping of M. tuberculosis Isolates

The Beijing genotype was the most distributed genotype among *M. tuberculosis* isolates in Kazakhstan and was identified in 65.2% (495/759) of cases among all TB cases; in groups with new and recurrent cases, it was found in 60% (324/540) and 78% (171/219) of cases, respectively. Non-Beijing genotypes of *M. tuberculosis* were determined in 34.8% (264/759) of all TB cases; among new and recurrent cases, 40% (216/540) and 22% (48/219) of patients had non-Beijing genotypes, respectively.

The results of RD typing using real-time PCR displayed that all 495 Beijing genotype isolates had the RD105 deletion, confirming their belonging to the Beijing family.

### 3.4. Clusters of the Beijing Genotype in Kazakhstan and Their Structure of Drug Resistance

In total, 119 MIRU-VNTR profile variants were identified among 495 Beijing genotype isolates across all cases using the 24 loci; 31 digital profiles were grouped into clusters comprising 2-260 clinical isolates according to the MIRU-VNTRplus nomenclature and accounted for 82.2% of Beijing genotype samples (407/495) (Table 3). The remaining 17.8% (88/495) of Beijing isolates revealed unique MIRU-VNTR profiles (Table S1).

According to the MLVA MtbC 15-9 classification, six major clusters were identified among Beijing genotype isolates, which accounted for 65.2% of all Beijing samples (323/495). Central Asian/Russian cluster 94-32 was the most distributed Beijing cluster in Kazakhstan (52.5%). The other five dominant Beijing clusters were found in 12.7% of cases: 96-32 (3.03%), 94-33 (2.82%), 100-32 (2.42%), 96-145 (2.22%) and 99-32 (2.22%). Eight isolates (1.62%) were classified as MLVA MtbC 15-9 type 95-32. Clusters 97-32 and 94-15 each had seven (1.41%) isolates; clusters 7188-32 and 7308-32 each contained six (1.21%) samples. MLVA MtbC 15-9 types 1047-32 and 1076-32 each consisted of four samples (0.81%). Six clusters (11427-32, 9344-32, 9343-32, 1048-32, 1075-32 and 7553-32) each included three (0.61%) isolates, and twelve clusters (11460-32, 11776-32, 4402-32, 1068-32, 95-33, 9126-32, 809-32, 9342-32, 94-554, 94-145, 99-15 and ?-32) each had two (0.4%) isolates of the Beijing genotype (Table 3).

In total, 79.9% (325/407) of clustered Beijing isolates were drug-resistant and had one of the resistant TB forms (mono- or polyresistant TB, MDR-TB); the remaining 20.1% (82/407) of samples were susceptible to the first-line anti-TB drugs used in treatment (Table 3).

The number of clustered samples did not differ significantly among Beijing genotype isolates from new (79.3%) and recurrent cases (77.2%). However, according to the MIRU-VNTRplus nomenclature, among clustered samples, 2.3 times more MLVA MtbC types 15-9 were identified in TB patients with new cases (23 clusters) compared to recurrent cases (10 clusters).

Six clusters, namely 94-32, 96-32, 94-33, 100-32, 96-145 and 99-32, were identified in 65.2% (323/495) of samples and were the dominant clusters among Beijing genotype isolates in our sample collection.

Table S2 displays characteristics of TB patients with different *M. tuberculosis* genotypes, including various Beijing clusters among all TB cases.

In our study, MDR samples predominated among all cases (41.4%); isolates with other DR and susceptible samples accounted for 22.7% and 35.9%, respectively. Overall, MDR-TB was more common among TB patients with the Beijing genotype (54.55%) than among those with non-Beijing genotypes (16.7%). Isolates with other DR (mono- and/or polyresistant) were detected in almost equal quantities among individuals with the Beijing genotype (22.62%) and non-Beijing genotypes (22.7%). Patients with susceptible TB mostly had non-Beijing genotypes (60.6%) compared to Beijing genotypes (22.83%) (Table S2).

### 3.5. Association of M. tuberculosis Beijing Genotype and Its Dominant Clusters with Drug Resistance in Kazakhstan

Statistical analysis of the data among all TB cases (*n* = 759) showed that the Beijing genotype was almost two times more common among drug-resistant isolates compared to non-Beijing genotypes (77.2% vs. 39.4%; *p* < 0.0001). Moreover, the Beijing genotype was associated with MDR-TB rather than with other DR forms of TB (70.7% vs. 29.3%; *p* < 0.0001) (Table 4). Across groups, among both new and recurrent TB cases, the Beijing genotype was also significantly associated with drug resistance (*p* < 0.0001 and *p* < 0.0001, respectively), including MDR-TB (*p* < 0.0001 and *p* = 0.0118, respectively) (Table 5).

The most distributed Central Asian/Russian cluster 94-32 (52.5%; *n* = 260) was significantly associated with DR-TB (*p* = 0.010), but not with MDR-TB (*p* = 0.070), among all cases (Table 4). In terms of groups, cluster 94-32 was not associated with antibiotic resistance among new TB cases (*p* = 0.149), while isolates of cluster 94-32 obtained from recurrent cases confirmed the association with drug resistance (*p* = 0.0191; OR = 4.8958; 95%CI: 1.2965–18.4883). This is probably due to the fact that resistant isolates were more prevalent among recurrent than new cases (*p* < 0.0001). However, when resistant samples were divided into MDR and other DR groups among recurrent cases, there was no association between 94-32 and any form of DR. On the contrary, a statistically significant association was identified among new TB cases between cluster 94-32 and MDR-TB; among new TB cases, this cluster was almost two times more frequently isolated from patients with MDR-TB than from patients with other forms of DR (66.4% vs. 33.6%) (*p* = 0.021) (Table 5).

The Russian cluster 100-32, found in 2.42% of our study’s *M. tuberculosis* Beijing population, did not show an association with DR-TB, including MDR-TB. Other dominant Beijing clusters such as 99-32 (2.22%), 94-33 (2.82%), 96-32 (3.03%) and 96-145 (2.22%) also were not associated with any form of drug resistance (Table 4). In terms of groups, among new and recurrent cases, similar results were obtained as for the group with all cases, and clusters 100-32, 99-32, 94-33, 96-32 and 96-145 had no association with resistance or susceptible TB.

Among the remaining 17% (84/495) of clustered Beijing isolates, 25 clusters were identified (Table 3). Statistical analysis of the data showed that cluster 94-15, compared with other clusters of the Beijing genotype, was associated with drug-susceptible TB infection (71.4% vs. 28.6%; *p* = 0.0100; OR = 8.7963; 95%CI: 1.6831–45.9730) (Table 6). Across groups, cluster 94-15 showed an association only with recurrent TB cases and not with new TB cases (Table 5).

It is important to note that in our study, among non-Beijing isolates, the LAM genotype displayed an association with drug resistance among all cases. Among LAM isolates (*n* = 95), there were 1.9 times more resistant samples in comparison to non-LAM isolates (all other genotypes, except Beijing combined into one group) (*n* = 169) (56.8% vs. 29.6%; *p* < 0.0001). Among drug-resistant samples, MDR isolates were 1.8 times more frequently distributed among LAM isolates in contrast with non-LAM isolates (53.7% vs. 30%; *p* = 0.018).

Regarding groups, the association of LAM genotype with drug resistance was observed among new cases (*p* < 0.0001) and was not detected among recurrent TB cases. Among drug-resistant isolates, LAM genotype was not associated with MDR-TB in groups of isolates from new and recurrent cases (Table 5).

Comparative analysis of statistically significant results among new and recurrent TB cases: 

A comparative analysis of data from clinical isolates of *M. tuberculosis* gathered from new and recurrent cases showed statistically significant results, displaying an association of the Beijing genotype with recurrent TB cases in Kazakhstan (*p* < 0.0001). Also in our study, drug-resistant forms of TB, including MDR-TB, were significantly associated with recurrent rather than new TB cases (*p* < 0.0001).

### 3.6. Association of Beijing Genotype and Its Dominant Clusters with Clinical Forms of Tuberculosis in Kazakhstan

In our study, infiltrative pulmonary TB (IPT) was the main clinical form of TB that was diagnosed in all groups—among TB patients with Beijing genotype (86.3%), including Beijing clusters 94-32 (86.9%), 96-32 (86.6%), 94-33 (78.6%), 100-32 (91.7%), 99-32 (90.9%) and other Beijing (86.6%), as well as other non-Beijing genotypes (86.4%) (Table S2). Despite that, there were two times more clinical forms of TB among new cases in comparison to recurrent cases; IPT was the most common clinical form of TB in both cases.

Statistical analysis of the association between the Beijing genotype and its dominant clusters (94-32, 99-32, 94-33, 96-32, 96-145, and 100-32) and clinical forms of TB was conducted.

Association analysis of *M. tuberculosis* genotypes and different clinical forms of TB determined that patients with tuberculous pleurisy had 2.7 times more non-Beijing genotypes than the Beijing genotype among all cases (3.8% vs. 1.4%; *p* = 0.0430; OR = 0.3643; 95%CI: 0.1371–0.9686) (Table S3). Regarding groups, a tendency was identified for the association of tuberculous pleurisy with non-Beijing genotypes among new cases; among recurrent cases, this relationship was not found (Table 5).

Among the main clusters of Beijing genotype among all TB cases, a relationship of the primary TB complex with clusters 94-33 (*p* = 0.0204; OR = 18.4231; 95%CI: 1.5694–216.2672) and 96-145 (*p* = 0.0119; OR = 24.1000; 95%CI: 2.0165–288.0242) was observed. However, it is worth noting that among all cases there were only three patients with primary TB complex (Table S4), and overall this clinical form of TB did not determine the association with the Beijing genotype (Table S3). There were no patients with primary TB complex among recurrent cases. Therefore, a relationship between the primary TB complex and clusters 94-33 (9.1% vs. 0.6%; *p* = 0.0302; OR = 15.5500; 95%CI: 1.3000–186.0055) and 96-145 (16.7% vs. 0.6%; *p* = 0.0081; OR = 31.6000; 95%CI: 2.4480–407.9163) was observed among new TB cases. In order to confirm the relationship of Beijing clusters 94-33 and 96-145 with primary TB complex in the future, more samples with this clinical form of TB should be analyzed.

Statistical analysis of the Beijing cluster 99-32 in comparison with other Beijing isolates did not show an association of any Beijing cluster with tuberculous pleurisy and cirrhotic TB among all cases (*p* > 0.05) (Table S4). However, when the analysis was conducted in groups of new and recurrent TB cases, we observed that tuberculous pleurisy and cirrhotic TB were associated with other Beijing isolates, rather than with cluster 99-32, among recurrent cases (*p* < 0.05). This result was most likely influenced by the fact that among recurrent cases there were no patients with tuberculous pleurisy and cirrhotic TB that had isolates of the cluster 99-32, and there was only one patient with each of these clinical forms of TB, who had the non-99-32 Beijing cluster (in both cases the isolate that was included in the group ‘other Beijing’ belonged to the Beijing cluster 94-32). Among new cases, there were no patients with cirrhotic TB, and in the case of tuberculous pleurisy, the association with other Beijing isolates (non-99-32) was not determined.

### 3.7. Association of Clinical Forms of TB with Drug Resistance

To investigate which clinical form of TB is associated with drug-resistant strains of *M. tuberculosis*, we additionally performed an association analysis of various clinical forms of TB and drug resistance.

Statistical analysis of clinical forms of TB with various forms of drug resistance in our research showed a significant association of fibrous–cavernous tuberculosis (FCT) with resistant *M. tuberculosis* isolates among all cases (*n* = 759) (*p* = 0.0019; OR = 3.3801; 95%CI: 1.5692–7.2807). Among resistant isolates, a relationship was found between FCT and MDR-TB (*p* = 0.0054; OR = 3.2453; 95%CI: 1.4167–7.4345) (Table 7). However, in terms of groups, there was no association between FCT and resistant *M. tuberculosis* isolates, including MDR *M. tuberculosis* isolates, among both new (*n* = 540) and recurrent cases (*n* = 219) (Table 4).

Among all cases (*n* = 759), disseminated TB was not associated with drug-resistant TB, in contrast to susceptible TB. But when drug-resistant isolates were divided into groups of MDR isolates and isolates with other DR (mono- and/or polyresistant TB), an association of disseminated TB with other DR was observed (*p* = 0.0295; OR = 0.0407; 95%CI: 0.0023–0.7274) (Table 7). In terms of groups, disseminated TB was not diagnosed among recurrent cases. Among new cases, there was a tendency for the relationship between this clinical form of TB and other DR to arise (4.2% vs. 0%; *p* = 0.0666; OR = 0.0672; 95%CI: 0.0037–1.2030).

Distribution of susceptible strains of *M. tuberculosis* was observed among patients with focal TB more than among patients with other diagnoses, and this relationship was statistically significant (*p* = 0.0072; OR = 0.1212; 95%CI: 0.0260–0.5651) (Table 7). Regarding groups, focal TB was associated with susceptible TB among new cases (*p* = 0.0254; OR = 0.1723; 95%CI: 0.0369–0.8050), and among recurrent cases, there were no patients with focal TB (Table 5).

Other clinical forms of TB, such as IPT, tuberculous pleurisy, caseous pneumonia, cirrhotic TB, primary TB complex, and miliary TB, did not show statistically significant associations with drug-resistant TB, including MDR-TB or drug-susceptible TB (Table 7). In terms of groups, both among new and recurrent TB cases, no statistically significant relationships were identified between the mentioned clinical forms of TB and different DR types.

## 4. Discussion

The Beijing genotype is the most widespread *M. tuberculosis* genotype in Kazakhstan. Here, we investigated the association of drug resistance (including MDR) and clinical forms of tuberculosis with the Beijing genotype and its dominant clusters, and compared obtained results among new, recurrent and all TB cases (new + recurrent TB cases) for the first time in Kazakhstan.

In this study, DST results revealed the prevalence of drug-resistant isolates among all TB cases (64%; 486/759): 64.6% of drug-resistant samples were MDR. DR isolates, including MDR isolates, were most commonly detected among recurrent TB cases (*p* < 0.0001), as expected. According to the WHO statistics, drug-resistant TB, in particular MDR/RR-TB, is 5.4 times more common among recurrent cases than among new TB cases (17.7% vs. 3.3%), and more than 50% of recurrent TB cases are registered in the former USSR countries [27]. The high frequency of MDR isolates in our sample collection confirms the ongoing trend of increasing MDR-TB, which is specific to Kazakhstan and to countries in Central Asia and Eastern Europe [27].

Among monoresistant *M. tuberculosis* isolates, resistance to streptomycin was primarily detected. This most likely indicates unsystematic and uncontrolled use of the drug and disruption of the continuity of treatment for other infectious diseases.

In this study, the Beijing genotype of *M. tuberculosis* was identified in 65.2% (495/759) of all TB cases based on 24 MIRU-VNTR analysis. All Beijing genotype isolates (*n* = 495) had a deletion of the RD105 locus. Currently, RD105 locus deletion is a marker of the Beijing genotype [22] and is used to identify Beijing isolates [28].

In our study, the Beijing genotype was associated with drug-resistant TB among both new and recurrent TB cases (*p* < 0.0001 and *p* < 0.0001, respectively), including MDR-TB (*p* < 0.0001 and *p* = 0.0118, respectively). In countries of the Commonwealth of Independent States (CIS), a relationship between the Beijing genotype and drug-resistant TB, including MDR-TB, has also been observed [29,30,31]. According to studies, MDR isolates of the Beijing genotype from Russia showed high virulence against macrophage-like cells [32], which may explain the high adaptability of Beijing strains for spreading in this region.

Among the Beijing genotype, six main clusters were identified, 94-32 (52.5%), 96-32 (3.03%), 94-33 (2.82%), 100-32 (2.42%), 96-145 (2.22%) and 99-32 (2.22%), which accounted for 65.2% (323/495) of all Beijing isolates in Kazakhstan. All these Beijing clusters, except cluster 96-145, were previously detected in the Former Soviet Union countries [14,33].

The Central Asian/Russian cluster 94-32 is the most distributed cluster of the Beijing genotype in our study (52.5%; 260/495). This cluster is also one of the largest Beijing clusters found in other Central Asian countries such as Uzbekistan, Tajikistan, and Kyrgyzstan [14]. The high prevalence of the Central Asian/Russian cluster 94-32 isolates in Central Asia, including Kazakhstan, underscores the need for further research on this Beijing cluster to improve measures to control its spread.

Central Asian/Russian cluster 94-32 was associated with drug-resistant *M. tuberculosis* isolates among all TB cases and recurrent cases, as well as with MDR-TB among new cases. In neighboring Russia, MDR was correlated with the Beijing B0/W148 subgroup (mostly cluster 100-32) compared with the Central Asian/Russian subgroup (mostly cluster 94-32) (*p* < 0.0001) [33]. In the study by Engstrom et al. [14], among isolates collected from three Central Asian countries (Uzbekistan, Tajikistan, and Kyrgyzstan), the Central Asian/Russian cluster 94-32 had a drug resistance pattern similar to that of other Beijing isolates.

The Russian cluster 100-32 in our study was found in only 2.42% (12/495) of cases. The Beijing 100-32 isolates are the main component of the epidemic subgroup B0/W148, which was significantly associated with MDR-TB in Russia [33]. In a recent study [14], Engstrom et al. revealed that the Russian cluster 100-32 was the second most important Beijing cluster spread among *M. tuberculosis* isolates from the three Central Asian countries at 11.5% (according to distribution in countries: Uzbekistan—5.1%; Tajikistan—24.8%; and Kyrgyzstan—4.2%). The smaller number of Beijing 100-32 isolates in Kazakhstan compared with other Central Asian republics [14] may suggest its recent emergence in the country.

In our work, only 50% of the Russian cluster 100-32 samples were multidrug-resistant. However, most Beijing 100-32 isolates from other Central Asian countries (Uzbekistan, Tajikistan, and Kyrgyzstan) were MDR, pre-XDR, or XDR (66/70; 94.3%) [14].

In our study, 1.41% (7/495) of Beijing isolates belonged to cluster 94-15, which was associated with drug-susceptible TB among all TB cases and recurrent TB cases (*p* < 0.05). It is suggested that the cluster 94-15 is a recently formed local clone in Poland. Polish samples of the Beijing genotype were assigned to cluster 94-32 or 94-15 in ~75% (18/25) cases, with digital profiles that differ only at the MIRU39 locus (three and two repeats, respectively). Almost all Beijing isolates (24/25; 96%) in that study were MDR or pre-XDR [34]. In a recent study of isolates collected from six regions of Northwestern Russia, cluster 94-15 was not identified [33]. The distribution of the Beijing cluster 94-15 in Kazakhstan, identified among *M. tuberculosis* isolates from Poland, was likely driven by trade and economic relations between Kazakhstan and the European Union, including Poland.

In the future, cross-border and local transmission networks of the Beijing clusters, specifically Central Asian/Russian cluster 94-32, Russian cluster 100-32 and cluster 94-15, need to be studied by a method with a higher resolution, such as whole-genome sequencing, as the MIRU-VNTR method has a limited resolution.

Among non-Beijing isolates, the LAM genotype was associated with drug resistance (among new cases and all TB cases), including MDR (among all TB cases) (*p* < 0.05). LAM isolates were associated with MDR-TB in Russia too [30]. This demonstrates that among non-Beijing isolates, the LAM genotype plays an important role in the distribution of MDR-TB in our region.

Among clinical forms of TB, fibrous–cavernous TB revealed a statistically significant association with drug resistance, including MDR, among all TB cases (*p* < 0.05). FCT was the second most frequently diagnosed clinical form of TB among patients after infiltrative pulmonary tuberculosis (7%; 53/759), and among patients with FCT, DR isolates were identified in 84.9% (45/53) of cases; 71.7% (38/53) of these isolates were MDR. According to published data, MDR-TB or XDR-TB is mostly diagnosed in patients with FCT [35].

Disseminated TB was not associated with DR overall among all TB cases; however, when DR isolates were divided into groups of samples with MDR and other DR, a relationship with other DR was found (*p* = 0.0295). This can be explained by the fact that all DR isolates (*n* = 6; 100%) collected from patients with disseminated TB had mono- and/or polyresistance.

Focal TB was associated with drug-susceptible isolates among patients with new TB cases (*p* = 0.0072), which may be because *M. tuberculosis* isolates from recurrent cases are predominantly resistant to antibiotics. In our study, focal TB was diagnosed in 1.44% (11/759) of cases, and in 81.8% (9/11) of cases, patients with focal TB had susceptible *M. tuberculosis* isolates.

Among all TB cases, a statistically significant association was found between tuberculous pleurisy and infection with non-Beijing genotypes compared with the Beijing genotype (*p* = 0.0430). Overall, 58.8% (10/17) of samples isolated from patients with tuberculous pleurisy were classified as non-Beijing genotypes.

An association of the Beijing genotype with fibrous–cavernous TB, disseminated TB, and caseous pneumonia was found in Russia [36]. Furthermore, the association between clinical forms of TB and a specific subgroup of Beijing genotype has been demonstrated. Thus, in Irkutsk (Eastern Siberia), chronic patients with FCT mostly had the Beijing B0/W148 subgroup. In the same study, among patients with disseminated TB (who were co-infected with HIV), the B0/W148 subgroup was detected more frequently than other genotypes [37]. In our study, almost all patients with the Beijing 100-32 genotype (91.7%; 11/12) were diagnosed with infiltrative pulmonary tuberculosis, and only one (8.3%; 1/12) had FCT. In our research, disseminated TB, FCT, and caseous pneumonia did not show a statistically significant association with the Beijing genotype and its main clusters, either among all TB cases or among new and recurrent cases. Interestingly, in a recent publication [36], disseminated TB showed no correlation with a specific *M. tuberculosis* genotype in the Omsk region (Western Siberia).

Limitations of the study: This study has several limitations that warrant acknowledgement. A primary limitation was the lack of comprehensive data on confounders. We did not consider some variables, including clinical factors (comorbidities, etc.), behavioral factors, and other variables that could have influenced the observed associations. Secondly, we performed multiple association analyses. Multiple testing, in turn, increases the probability of Type I Error. Although we applied statistical methods with corrections, we cannot rule out the possibility of false-positive results. Furthermore, 86.3% of the TB patients were diagnosed with infiltrative pulmonary tuberculosis, and the remaining eight clinical forms of TB were detected in small quantities (0.13–7%). Sparse clinical categories with limited sample sizes reduce statistical power and may lead to unstable estimates with wide confidence intervals. Consequently, the restricted representation of certain clinical forms of TB limits the generalizability of the findings. Lastly, the 24-locus MIRU-VNTR genotyping method used in the study has high discriminatory capacity. However, whole-genome sequencing provides higher resolution for investigating cross-border and local transmission networks and should be performed in future studies for these purposes.

All listed study limitations highlight the need for cautious interpretation of the results and underline the importance of future studies with larger, more diverse samples and improved data completeness.

## 5. Conclusions

The *M. tuberculosis* Beijing genotype, particularly the Central Asian cluster 94-32, plays a significant role in the distribution of drug-resistant TB among all TB cases and recurrent cases, including MDR-TB among new TB cases in Kazakhstan. On the contrary, the Beijing cluster 94-15 may contribute to the spread of drug-susceptible TB among all TB cases and recurrent TB cases in the country. That is why further investigation of the genomic features of these *M. tuberculosis* Beijing clusters, as well as their virulence and pathogenicity, is required.

## Figures and Tables

**Table 1:** Clinical characteristics of 759 TB patients among new and recurrent cases.

Clinical Forms of TB (Diagnosis)	New Cases [12]	Recurrent Cases	All Cases
Caseous pneumonia	2 (0.4%)	0	2 (0.3%)
Cirrhotic tuberculosis	0	1 (0.45%)	1 (0.13%)
Disseminated tuberculosis	14 (2.6%)	0	14 (1.8%)
Fibrous–cavernous tuberculosis	13 (2.4%)	40 (18.3%)	53 (7%)
Focal pulmonary tuberculosis	11 (2%)	0	11 (1.44%)
Infiltrative pulmonary tuberculosis	478 (88.5%)	177 (80.8%)	655 (86.3%)
Miliary tuberculosis	3 (0.55%)	0	3 (0.4%)
Primary tuberculosis complex	3 (0.55%)	0	3 (0.4%)
Tuberculous pleurisy	16 (3%)	1 (0.45%)	17 (2.23%)
Total:	540 (100%)	219 (100%)	759 (100%)

**Table 2:** Various combinations of drug resistance of clinical isolates of *M. tuberculosis*.

Drug-Resistant *M. tuberculosis* Isolates	New Cases [12]	Recurrent Cases	All Cases
Monoresistant (resistant to one drug only) isolates:	49 (16.3%)	12 (6.5%)	61 (12.6%)
resistant to RMP only	6 (2%)	2 (1.1%)	8 (1.6%)
resistant to INH only	14 (4.7%)	0	14 (2.9%)
resistant to EMB only	7 (2.3%)	3 (1.6%)	10 (2.1%)
resistant to SM only	22 (7.3%)	7 (3.8%)	29 (6%)
Multidrug resistant (at least resistant to RMP and INH) isolates:	157 (52.3%)	157 (84.4%)	314 (64.6%)
resistant to RMP + INH	13 (4.3%)	1 (0.5%)	14 (2.8%)
resistant to RMP + INH + EMB	3 (1%)	1 (0.5%)	4 (0.8%)
resistant to RMP + INH + SM	30 (10%)	31 (16.7%)	61 (12.5%)
resistant to RMP + INH + EMB + SM	111 (37%)	124 (66.7%)	235 (48.3%)
Polyresistant (resistant to two and more drugs, except RMP + INH combination) isolates:	94 (31.3%)	17 (9.1%)	111 (22.8%)
resistant to INH + EMB	4 (1.33%)	1 (0.53%)	5 (1%)
resistant to INH + SM	25 (8.32%)	7 (3.75%)	32 (6.6%)
resistant to INH + EMB + SM	28 (9.32%)	6 (3.21%)	34 (7%)
resistant to RMP + EMB	1 (0.33%)	1 (0.53%)	2 (0.4%)
resistant to RMP + SM	10 (3.32%)	1 (0.53%)	11 (2.3%)
resistant to RMP + EMB + SM	1 (0.33%)	0	1 (0.2%)
resistant to EMB + SM	25 (8.32%)	1 (0.53%)	26 (5.3%)
Total:	300 (100%)	186 (100%)	486 (100%)

RMP—rifampicin; INH—isoniazid; EMB—ethambutol; SM—streptomycin.

**Table 3:** Drug resistance of MLVA MtbC 15-9 types among clustered (2 or more isolates) *M. tuberculosis* clinical isolates of Beijing genotype among all cases.

MLVA MtbC 15-9 Types	24 MIRU-VNTR Profile	Drug-Resistant TB		Drug-Susceptible TB	Total Among All Beijing (*n* = 495)
		MDR-TB	Other DR-TB **		
94-32 *	244233352644425153353823	159 (61.1%)	54 (20.8%)	47 (18.1%)	260 (52.5%)
99-32 *	244233352644425153353723	4 (36.4%)	2 (18.1%)	5 (45.5%)	11 (2.22%)
11460-32	244233352644425153353223	1 (50%)	0	1 (50%)	2 (0.4%)
11427-32	244233352644425153353523	2 (66.7%)	1 (33.3%)	0	3 (0.61%)
9344-32	245233352644425153353823	3 (100%)	0	0	3 (0.61%)
11776-32	244233352244425153353823	1 (50%)	0	1 (50%)	2 (0.4%)
4402-32	244233352744425153353823	2 (100%)	0	0	2 (0.4%)
97-32	244233332644425153353823	3 (42.9%)	1 (14.2%)	3 (42.9%)	7 (1.41%)
9343-32	244234352644425153353823	0	2 (66.7%)	1 (33.3%)	3 (0.61%)
1068-32	244233352644425143353823	1 (50%)	0	1 (50%)	2 (0.4%)
1048-32	244233352644425173353823	0	3 (100%)	0	3 (0.61%)
94-15	244233352644425153353822	1 (14.3%)	1 (14.3%)	5 (71.4%)	7 (1.41%)
1047-32	244233252644425153353823	3 (75%)	0	1 (25%)	4 (0.81%)
94-33 *	244233352644425153353824	4 (28.55%)	4 (28.55%)	6 (42.9%)	14 (2.82%)
95-33	244233352634425153353824	1 (50%)	1 (50%)	0	2 (0.4%)
7188-32	244223352644425153353823	4 (66.7%)	2 (33.3%)	0	6 (1.21%)
9126-32	244233352624425153353823	2 (100%)	0	0	2 (0.4%)
7308-32	244232352644425153353823	4 (66.7%)	0	2 (33.3%)	6 (1.21%)
809-32	244232352634425153353823	0	1 (50%)	1 (50%)	2 (0.4%)
9342-32	244235352644425153353823	1 (50%)	1 (50%)	0	2 (0.4%)
95-32	244233352634425153353823	5 (62.5%)	3 (37.5%)	0	8 (1.62%)
1075-32	244233352644425163353723	2 (66.7%)	1 (33.3%)	0	3 (0.61%)
1076-32	244233352644425163353823	3 (75%)	1 (25%)	0	4 (0.81%)
96-32 *	244233362644425153353823	7 (46.6%)	4 (26.7%)	4 (26.7%)	15 (3.03%)
96-145 *	244233361644425153353823	9 (81.8%)	2 (18.2%)	0	11 (2.22%)
7553-145	244233361644425153343823	2 (66.7%)	1 (33.3%)	0	3 (0.61%)
94-554	244233351644425153353824	1 (50%)	1 (50%)	0	2 (0.4%)
94-145	244233351644425153353823	1 (50%)	1 (50%)	0	2 (0.4%)
99-15	244233352644425153353722	1 (50%)	1 (50%)	0	2 (0.4%)
100-32 *	244233352644425173353723	6 (50%)	4 (33.3%)	2 (16.7%)	12 (2.42%)
?-32	244232352544425153343823	0	0	2 (100%)	2 (0.4%)
Clustered isolates (2 and more isolates):		233 (57.3%)	92 (22.6%)	82 (20.1%)	407 (82.2%)
Isolates with individual MIRU-VNTR digital profiles (only 1 isolate):		37 (42.04%)	20 (22.73%)	31 (35.23%)	88 (17.8%)

* the most spread clusters (more than 10 isolates) in Kazakhstan; ** monoresistant and/or polyresistant TB was included in this group.

**Table 4:** Association of different *M. tuberculosis* genotypes, including Beijing clusters, with drug resistance among all cases.

Genotypes/Beijing Clusters	Drug-Resistant TB	Drug-Susceptible TB	OR	95%CI	*p*	MDR-TB	Other DR-TB **	OR	95%CI	*p*
Beijing non-Beijing	382 (77.2%) 104 (39.4%)	113 (22.8%) 160 (60.6%)	-	-	*p* < 0.0001 *	270 (70.7%) 44 (42.3%)	112 (29.3%) 60 (57.7%)	-	-	*p* < 0.0001 *
94-32 other Beijing	213 (81.9%) 169 (71.9%)	47 (18.1%) 66 (28.1%)	-	-	*p* = 0.010 *	159 (74.6%) 111 (65.7%)	54 (25.4%) 58 (34.3%)	-	-	*p* = 0.070
100-32 other Beijing	10 (83.3%) 372 (77%)	2 (16.7%) 111 (23%)	1.4919	0.3221–6.9103	*p* = 0.6090	6 (60%) 264 (71%)	4 (40%) 108 (29%)	0.6136	0.1698–2.2177	*p* = 0.4563
99-32 other Beijing	6 (54.5%) 376 (77.7%)	5 (45.5%) 108 (22.3%)	0.3447	0.1032–1.1512	*p* = 0.0834	4 (66.7%) 266 (70.7%)	2 (33.3%) 110 (29.3%)	0.8271	0.1493–4.5815	*p* = 0.8279
94-33 other Beijing	8 (57.1%) 374 (77.8%)	6 (42.9%) 107 (22.2%)	0.3815	0.1295–1.1234	*p* = 0.0803	4 (50%) 266 (71.1%)	4 (50%) 108 (28.9%)	0.4060	0.0997–1.6528	*p* = 0.2082
96-32 other Beijing	11 (73.3%) 371 (77.3%)	4 (26.7%) 109 (22.7%)	0.8080	0.2522–2.5880	*p* = 0.7196	7 (63.6%) 263 (70.9%)	4 (36.4%) 108 (29.1%)	0.7186	0.2061–2.5051	*p* = 0.6040
96-145 other Beijing	11 (100%) 371 (76.7%)	0113 (23.3%)	7.0269	0.4109–120.1830	*p* = 0.1783	9 (81.8%) 261 (70.4%)	2 (18.2%) 110 (29.6%)	1.8966	0.4032–8.9205	*p* = 0.4178

* statistically significant result; ** monoresistant and/or polyresistant isolates were included in this group.

**Table 5:** Statistically significant results of association studies among new cases, recurrent cases and all TB cases.

Hypothesis	*p*-Value		
	New Cases (*n* = 540)	Recurrent Cases (*n* = 219)	All Cases (*n* = 759)
Beijing genotype is associated with DR-TB	*p* < 0.0001 *	*p* < 0.0001 *	*p* < 0.0001 *
Beijing genotype is associated with MDR-TB	*p* < 0.0001 *	*p* = 0.0118 *	*p* < 0.0001 *
Cluster 94-32 is associated with DR-TB	*p* = 0.149	*p* = 0.0191 *	*p* = 0.010 *
Cluster 94-32 is associated with MDR-TB	*p* = 0.021 *	*p* = 0.3097	*p* = 0.070
Cluster 94-15 is associated with DS-TB **	*p* = 0.8682	*p* = 0.0283 *	*p* = 0.0100 *
Among non-Beijing isolates, LAM genotype is associated with DR-TB	*p* < 0.0001 *	*p* = 0.4077	*p* < 0.0001 *
Among non-Beijing isolates, LAM genotype is associated with MDR-TB	*p* = 0.0908	*p* = 0.0890	*p* = 0.018 *
Fibrous–cavernous TB is associated with DR-TB	*p* = 0.3220	*p* = 0.3267	*p* = 0.0019 *
Fibrous–cavernous TB is associated with MDR-TB	*p* = 0.1417	*p* = 0.7541	*p* = 0.0054 *
Focal TB is associated with DS-TB	*p* = 0.0254 *	0	*p* = 0.0072 *
Tuberculous pleurisy is associated with non-Beijing genotypes	*p* = 0.0714	*p* = 0.9230	*p* = 0.0430 *

* statistically significant result; ** drug-susceptible TB; 0—absence of cases with this form of TB.

**Table 6:** Association of other MLVA MtbC 15-9 Beijing *M. tuberculosis* types with drug resistance.

Beijing Clusters	Drug-Resistant TB	Drug-Susceptible TB	OR	95%CI	*p*	Beijing Clusters	MDR-TB	Other DR-TB **	OR	95%CI	*p*
99-15	2 (100%)	0	1.4915	0.0711–31.2932	*p* = 0.7968	11776-32	1 (100%)	0	1.2523	0.0506–30.9761	*p* = 0.8907
Other Beijing	380 (77.1%)	113 (22.9%)				Other Beijing	269 (70.6%)	112 (29.4%)			
94-145	2 (100%)	0	1.4915	0.0711–31.2932	*p* = 0.7968	4402-32	2 (100%)	0	2.0950	0.0998–43.9885	*p* = 0.6340
Other Beijing	380 (77.1%)	113 (22.9%)				Other Beijing	268 (70.5%)	112 (29.5%)			
94-554	2 (100%)	0	1.4915	0.0711–31.2932	*p* = 0.7968	11460-32	1 (100%)	0	1.2523	0.0506–30.9761	*p* = 0.8907
Other Beijing	380 (77.1%)	113 (22.9%)				Other Beijing	269 (70.6%)	112 (29.4%)			
7553-145	3 (100%)	0	2.0935	0.1073–40.8338	*p* = 0.6259	11427-32	2 (66.7%)	1 (33.3%)	0.8284	0.0743–9.2291	*p* = 0.8783
Other Beijing	379 (77%)	113 (23%)				Other Beijing	268 (70.7%)	111 (29.3%)			
11427-32	3 (100%)	0	2.0935	0.1073–40.8338	*p* = 0.6259	9344-32	3 (100%)	0	2.9439	0.1508–57.4640	*p* = 0.4763
Other Beijing	379 (77%)	113 (23%)				Other Beijing	267 (70.4%)	112 (29.6%)			
9344-32	3 (100%)	0	2.0935	0.1073–40.8338	*p* = 0.6259	97-32	3 (75%)	1 (25%)	1.2472	0.1283–12.1206	*p* = 0.8490
Other Beijing	379 (77%)	113 (23%)				Other Beijing	267 (70.6%)	111 (29.4%)			
4402-32	2 (100%)	0	1.4915	0.0711–31.2932	*p* = 0.7968	1068-32	1 (100%)	0	1.2523	0.0506–30.9761	*p* = 0.8907
Other Beijing	380 (77.1%)	113 (22.9%)				Other Beijing	269 (70.6%)	112 (29.4%)			
7308-32	4 (66.7%)	2 (33.3%)	0.5873	0.1062–3.2489	*p* = 0.5420	1048-32	0 (0%)	3 (100%)	0.0578	0.0030–1.1290	*p* = 0.0601
Other Beijing	378 (77.3%)	111 (22.7%)				Other Beijing	270 (71.2%)	109 (28.8%)			
9343-32	2 (66.7%)	1 (33.3%)	0.5895	0.0530–6.5614	*p* = 0.6673	1047-32	3 (100%)	0	2.9439	0.1508–57.4640	*p* = 0.4763
Other Beijing	380 (77.2%)	112 (22.8%)				Other Beijing	267 (70.4%)	112 (29.6%)			
1048-32	3 (100%)	0	2.0935	0.1073–40.8338	*p* = 0.6259	9126-32	2 (100%)	0	2.0950	0.0998–43.9885	*p* = 0.6340
Other Beijing	379 (77%)	113 (23%)				Other Beijing	268 (70.5%)	112 (29.5%)			
1047-32	3 (75%)	1 (25%)	0.8865	0.0913–8.6071	*p* = 0.9173	809-32	0 (0%)	1 (100%)	0.1374	0.0056–3.3987	*p* = 0.2253
Other Beijing	379 (77.2%)	112 (22.8%)				Other Beijing	270 (70.9%)	111 (29.1%)			
95-33	2 (100%)	0	1.4915	0.0711–31.2932	*p* = 0.7968	1075-32	2 (66.7%)	1 (33.3%)	0.8284	0.0743–9.2291	*p* = 0.8783
Other Beijing	380 (77.1%)	113 (22.9%)				Other Beijing	268 (70.7%)	111 (29.3%)			
9126-32	2 (100%)	0	1.4915	0.0711–31.2932	*p* = 0.7968	1076-32	3 (75%)	1 (25%)	1.2472	0.1283–12.1206	*p* = 0.8490
Other Beijing	380 (77.1%)	113 (22.9%)				Other Beijing	267 (70.6%)	111 (29.4%)			
9342-32	2 (100%)	0	1.4915	0.0711–31.2932	*p* = 0.7968	7553-145	2 (66.7%)	1 (33.3%)	0.8284	0.0743–9.2291	*p* = 0.8783
Other Beijing	380 (77.1%)	113 (22.9%)				Other Beijing	268 (70.7%)	111 (29.3%)			
1075-32	3 (100%)	0	2.0935	0.1073–40.8338	*p* = 0.6259	95-32	5 (62.5%)	3 (37.5%)	0.6855	0.1610–2.9186	*p* = 0.6095
Other Beijing	379 (77%)	113 (23%)				Other Beijing	265 (70.9%)	109 (29.1%)			
1076-32	4 (100%)	0	2.6988	0.1442–50.5077	*p* = 0.5065	7308-32	4 (100%)	0	3.7992	0.2028–71.1576	*p* = 0.3719
Other Beijing	378 (77%)	113 (23%)				Other Beijing	266 (70.4%)	112 (29.6%)			
94-15	2 (28.6%)	5 (71.4%)	8.7963	1.6831–45.9730	*p* = 0.0100 *	7188-32	4 (66.7%)	2 (33.3%)	0.8271	0.1493–4.5815	*p* = 0.8279
Other Beijing	380 (77.9%)	108 (22.1%)				Other Beijing	266 (70.7%)	110 (29.3%)			
?-32	0	2 (100%)	17.1525	0.8174–359.9191	*p* = 0.0672	-	-	-	-	-	-
Other Beijing	382 (77.5%)	111 (22.5%)				-	-	-	-	-	-

* statistically significant result; ** monoresistant and/or polyresistant isolates were included in this group.

**Table 7:** Association of clinical forms of TB with drug resistance among all cases (*n* = 759).

Clinical Forms of TB (Diagnosis)	Drug-Resistant TB	Drug-Susceptible TB	OR	95%CI	*p*	MDR-TB	Other DR-TB **	OR	95%CI	*p*
Disseminated TB Other clinical forms of TB	6 (1.2%) 480 (98.8%)	8 (2.9%) 265 (97.1%)	0.4141	0.1422–1.2061	0.1060	0314 (100%)	6 (3.5%) 166 (96.5%)	0.0407	0.0023–0.7274	0.0295 *
Fibrous–cavernous TB Other clinical forms of TB	45 (9.3%) 441 (90.7%)	8 (2.9%) 265 (97.1%)	3.3801	1.5692–7.2807	0.0019 *	38 (12.1%) 276 (87.9%)	7 (4.1%) 165 (95.9%)	3.2453	1.4167–7.4345	0.0054 *
Focal pulmonary TB Other clinical forms of TB	2 (0.4%) 484 (99.6%)	9 (3.3%) 264 (96.7%)	0.1212	0.0260–0.5651	0.0072 *	1 (0.3%) 313 (99.7%)	1 (0.6%) 171 (99.4%)	0.5463	0.0340–8.7897	0.6697
Infiltrative pulmonary TB Other clinical forms of TB	415 (85.4%) 71 (14.6%)	240 (87.9%) 33 (12.1%)	-	-	0.379	262 (83.4%) 52 (16.6%)	153 (89%) 19 (11%)	-	-	0.108
Tuberculous pleurisy Other clinical forms of TB	10 (2.1%) 476 (97.9%)	7 (2.6%) 266 (97.4%)	0.7983	0.3004–2.1217	0.6515	6 (1.9%) 308 (98.1%)	4 (2.3%) 168 (97.7%)	0.8182	0.2277–2.9400	0.7585
Caseous pneumonia Other clinical forms of TB	2 (0.4%) 484 (99.6%)	0273 (100%)	2.8225	0.1350–59.0071	0.5035	2 (0.6%) 312 (99.4%)	0172 (100%)	2.7600	0.1317–57.8201	0.5130
Cirrhotic TB Other clinical forms of TB	1 (0.2%) 485 (99.8%)	0273 (100%)	1.6900	0.0686–41.6304	0.7482	1 (0.3%) 313 (99.7%)	0172 (100%)	1.6507	0.0669–40.7422	0.7593
Primary TB complex Other clinical forms of TB	3 (0.6%) 483 (99.4%)	0273 (100%)	3.9597	0.2038–76.9465	0.3633	2 (0.6%) 312 (99.4%)	1 (0.6%) 171 (99.4%)	1.0962	0.0987–12.1769	0.9404
Miliary TB Other clinical forms of TB	2 (0.4%) 484 (99.6%)	1 (0.4%) 272 (99.6%)	1.1240	0.1014–12.4529	0.9241	2 (0.6%) 312 (99.4%)	0172 (100%)	2.7600	0.1317–57.8201	0.5130

* statistically significant result; ** monoresistant and/or polyresistant isolates were included in this group.

## Data Availability

The original contributions presented in this study are included in the article/Supplementary Materials. Further inquiries can be directed to the corresponding authors.

## Supplementary Materials

The following supporting information can be downloaded at https://www.mdpi.com/article/10.3390/microorganisms14092121/s1, Table S1 Drug resistance of MLVA MtbC 15-9 types among *M. tuberculosis* isolates of Beijing genotype with unique MIRU-VNTR digital profiles (only one isolate) among all cases; Table S2 Characteristics of TB patients with different *M. tuberculosis* genotypes, including various Beijing clusters among all TB cases; Table S3 Association of different *M. tuberculosis* genotypes, including Beijing clusters 94-32 and 100-32 with clinical forms of TB among all cases (*n* = 759); Table S4 Association of Beijing clusters 99-32, 94-33, 96-32 and 96-145 with clinical forms of TB among all cases (*n* = 759).
