# Supplementary material for: M. tuberculosis Beijing Genotype Clusters: Impact on Tuberculosis Drug Resistance in Kazakhstan
Source: Microorganisms. 2026 Sep 21;14(9):2121. doi: 10.3390/microorganisms14092121 (PMC13609652; doi:10.3390/microorganisms14092121)
Supplement: Supplementary file 1 [file microorganisms-14-02121-s001.zip › microorganisms-4490052-supplementary.pdf]

Table S1. Drug resistance of MLVA MtbC 15-9 types among *M. tuberculosis* isolates of Beijing genotype with unique MIRU-VNTR digital profiles (only one isolate) among all cases

MLVA MtbC 15-9 types	24 MIRU-VNTR profile	Drug susceptibility profile	MLVA MtbC 15-9 types	24 MIRU-VNTR profile	Drug susceptibility profile
99-332	244233352642425153353723	susceptible	2057-32	244233352634425173353823	monoresistant
94-17	244233352644425153253822	susceptible	895-32	244233362644425173353823	monoresistant
96-1595	244233363644425153353824	polyresistant	3819-32	244233352534425173353823	monoresistant
11775-135	244233362644435143353823	susceptible	11793-32	242233362644425153363823	susceptible
96-572	244233362644425154353824	susceptible	95-34	244233352633425153353823	susceptible
783-32	244243352644425153353823	MDR	95-175	244233352634426153353823	monoresistant
783-122	244243352644425154353823	MDR	4737-32	244233352644425173343723	MDR
21601-32	234233352644425173343823	susceptible	18128-229	244233352644425152353423	polyresistant
10977-32	244233352644425173353123	MDR	10986-32	244233352644425153353123	MDR
407-32	244233352544425153353823	susceptible	18128-32	244233352644425153353423	polyresistant
9358-32	224233352644425153353823	susceptible	10523-145	244233351644425153353833	polyresistant
4398-32	244233352334425153353823	susceptible	801-33	244233342644425153353824	polyresistant
14655-32	244233352644425133353823	polyresistant	4402-795	244233352744427153353823	susceptible
9125-32	244233352642425153353923	polyresistant	96-15	244233362644425153353822	susceptible
17270-32	244233352644425153354623	MDR	20584-62	244233352144426153353822	susceptible
4499-32	244233352644425153352823	MDR	9356-32	242233352644425153363823	susceptible
3838-32	244233352644425173353523	MDR	9363-32	244233452644425153353823	MDR
20657-32	244233352644425113353823	MDR	3836-32	244233352644425173353223	susceptible
9124-32	234233352644425153353823	MDR	10773-32	244213352544425173353923	susceptible
407-15	244233352544425153353822	MDR	2224-32	244233352534425153353823	polyresistant
1069-145	244233351634425173353723	MDR	?-145	244233361644425153333833	MDR
9125-32	244233352644425153353923	MDR	?-145	242233361644425153353823	MDR
9129-33	244233352644425153343824	MDR	?-145	244233381644425153353823	monoresistant
9136-33	244243352644425153343724	susceptible	?-33	244233362644425113353824	MDR
22386-15	244233352644425153353712	MDR	?-33	242233362644425153353824	susceptible
11254-32	254233352644425173343723	MDR	?-32	244233152644425153363823	susceptible
9361-32	244233372644425153343823	susceptible	?-32	243235352644425163353823	polyresistant
4737-33	244233352644425173343724	MDR	?-32	242133352644425153363823	polyresistant
11778-32	244243352644425153353723	susceptible	?-32	244233352634425153354823	susceptible
1070-32	244223352644425173353723	MDR	?-32	214233352644425153354823	MDR
4743-32	244233252644425173353823	MDR	?-32	244233352334425153354823	susceptible
20584-98	244233352144425153253821	MDR	?-32	244232352144425153333823	susceptible
6827-32	244232352644425173353723	MDR	?-32	244233352544425143333823	susceptible
20584-32	244233352144425153353823	MDR	?-32	254233252644425173353823	MDR
12576-32	244233352644425153353803	MDR	?-32	254233352244125153353823	MDR
11469-32	244223352644425143353823	MDR	?-32	244235352644425153333823	susceptible
1065-32	244233352644425153353623	MDR	?-32	254223352644425153353823	susceptible
16400-32	2442333526444251533431023	MDR	?-32	244233382544425153354823	susceptible
10519-32	244233352644425143353723	susceptible	?-32	244243352634425143333823	MDR
230-32	243233352644425153353823	MDR	?-32	244233322634425153353123	polyresistant
94-25	244233352644425153253823	monoresistant	?-32	224233352144425153353833	polyresistant
1065-387	244233352645425153353623	MDR	?-32	244232352744425153353823	polyresistant
95-387	244233352635425153353823	susceptible	?-32	244243352624425153353823	MDR
9385-32	244233352544425153343823	susceptible	?-32	244234352544425163353823	polyresistant
Total number of samples:		44	Total number of samples:		44

Table S2. Characteristics of TB patients with different *M. tuberculosis* genotypes, including various Beijing clusters among all TB cases

***M. tuberculosis* isolates**	**Number of Beijing isolates**	**Beijing clusters**							**Number of non-Beijing isolates**	**Total**
		**94-32**	**99-32**	**94-33**	**96-32**	**96-145**	**100-32**	**other Beijing***		
Total	495 (65.2%)	260 (52.53%)	11 (2.22%)	14 (2.83%)	15 (3.03%)	11 (2.22%)	12 (2.42%)	172 (34.75%)	264 (34.8%)	759 (100%)
Clinical forms of TB (based on clinical and X-ray characteristics)										
Disseminated TB	9 (1.8%)	5 (1.9%)	0	1 (7.1%)	1 (6.7%)	0	0	2 (1.2%)	5 (1.9%)	14 (1.8%)
Fibrous-cavernous TB	40 (8.1%)	22 (8.5%)	0	1 (7.1%)	1 (6.7%)	2 (18.2%)	1 (8.3%)	13 (7.5%)	13 (4.9%)	53 (7%)
Focal pulmonary TB	4 (0.8%)	2 (0.8%)	0	0	0	0	0	2 (1.2%)	7 (2.6%)	11 (1.44%)
Infiltrative pulmonary TB	427 (86.3%)	226 (86.9%)	10 (90.9%)	11 (78.6%)	13 (86.6%)	7 (63.6%)	11 (91.7%)	149 (86.6%)	228 (86.4%)	655 (86.3%)
Tuberculous pleurisy	7 (1.4%)	3 (1.1%)	1 (9.1%)	0	0	1 (9.1%)	0	2 (1.2%)	10 (3.8%)	17 (2.23%)
Primary TB complex	3 (0.6%)	0	0	1 (7.1%)	0	1 (9.1%)	0	1 (0.5%)	0	3 (0.4%)
Miliary TB	2 (0.4%)	0	0	0	0	0	0	2 (1.2%)	1 (0.4%)	3 (0.4%)
Caseous pneumonia	2 (0.4%)	1 (0.4%)	0	0	0	0	0	1 (0.5%)	0	2 (0.3%)
Cirrhotic TB	1 (0.2%)	1 (0.4%)	0	0	0	0	0	0	0	1 (0.13%)
Drug-resistant TB:										
MDR-TB	270 (54.55%)	159 (61.1%)	4 (36.4%)	4 (28.6%)	7 (46.6%)	9 (81.8%)	6 (50%)	81 (47.1%)	44 (16.7%)	314 (41.4%)
other DR-TB**	112 (22.62%)	54 (20.8%)	2 (18.2%)	4 (28.6%)	4 (26.7%)	2 (18.2%)	4 (33.3%)	42 (24.4%)	60 (22.7%)	172 (22.7%)
Drug-susceptible TB	113 (22.83%)	47 (18.1%)	5 (45.4%)	6 (42.8%)	4 (26.7%)	0	2 (16.7%)	49 (28.5%)	160 (60.6%)	273 (35.9%)

* - this group consists of Beijing isolates except 94-32, 99-32, 94-33, 96-32, 96-145 and 100-32 ; ** - monoresistant and/or polyresistant isolates were included into this group

Table S3. Association of different *M. tuberculosis* genotypes, including Beijing clusters 94-32 and 100-32 with clinical forms of TB among all cases (n=759)

**Clinical forms of TB (diagnosis)**	**Beijing**	**non-Beijing**	**94-32**	**Other Beijing**	**100-32**	**Other Beijing**
Disseminated TB	9 (1.8%)	5 (1.9%)	5 (1.9%)	4 (1.7%)	0	9 (1.9%)
Other clinical forms of TB	486 (98.2%)	259 (98.1%)	255 (98.1%)	231 (98.3%)	12 (100%)	474 (98.1%)
Statistics	p=0.9411; OR=0.9593; 95%CI=0.3182-2.8921		p=0.8543; OR=1.1324; 95%CI=0.3004-4.2678		p=0.6398; OR=1.9979; 95%CI=0.1101-36.2662	
Fibrous-cavernous TB	40 (8.1%)	13 (4.9%)	22 (8.5%)	18 (7.7%)	1 (8.3%)	39 (8.1%)
Other clinical forms of TB	455 (91.9%)	251 (95.1%)	238 (91.5%)	217 (92.3%)	11 (91.7%)	444 (91.9%)
Statistics	p=0.134		p=0.869		p=0.9741; OR=1.0350; 95%CI=0.1302-8.2279	
Infiltrative pulmonary TB	427 (86.3%)	228 (86.4%)	226 (86.9%)	201 (85.5%)	11 (91.7%)	416 (86.1%)
Other clinical forms of TB	68 (13.7%)	36 (13.6%)	34 (13.1%)	34 (14.5%)	1 (8.3%)	67 (13.9%)
Statistics	p=1.0000		p=0.696		p=0.5870; OR=1.7716; 95%CI=0.2250-13.9468	
Focal pulmonary TB	4 (0.8%)	7 (2.7%)	2 (0.8%)	2 (0.9%)	0	4 (0.8%)
Other clinical forms of TB	491 (99.2%)	257 (97.3%)	258 (99.2%)	233 (99.1%)	12 (100%)	474 (99.2)
Statistics	p=0.0560; OR=0.2991; 95%CI=0.0868-1.0312		p=0.9191; OR=0.9031; 95%CI=0.1262-6.4628		p=0.3395; OR=4.2622; 95%CI=0.2175-83.5178	
Tuberculous pleurisy	7 (1.4%)	10 (3.8%)	3 (1.2%)	4 (1.7%)	0	7 (1.4%)
Other clinical forms of TB	488 (98.6%)	254 (96.2%)	257 (98.8%)	231 (98.3%)	12 (100%)	476 (98.6%)
Statistics	p=0.0430*; OR=0.3643; 95%CI=0.1371-0.9686		p=0.6082; OR=0.6741; 95%CI=0.1493-3.0439		p=0.5309; OR=2.5413; 95%CI=0.1374-46.9939	
Caseous pneumonia	2 (0.4%)	0	1 (0.4%)	1 (0.4%)	0	2 (0.4%)
Other clinical forms of TB	493 (99.6%)	264 (100%)	259 (99.6%)	234 (99.6%)	12 (100%)	481 (99.6%)
Statistics	p=0.5251; OR=2.6798; 95%CI=0.1282-56.0277		p=0.9429; OR=0.9035; 95%CI=0.0562-14.5266		p=0.1950; OR=7.7040; 95%CI=0.3513-168.9568	
Cirrhotic TB	1 (0.2%)	0	1 (0.4%)	0	0	1 (0.2%)
Other clinical forms of TB	494 (99.8%)	264 (100%)	259 (99.6%)	235 (100%)	12 (100%)	482 (99.8%)
Statistics	p=0.7724; OR=1.6047; 95%CI=0.0651-39.5298		p=0.5403; OR=2.7225; 95%CI=0.1104-67.1608		p=0.1234; OR=12.8667; 95%CI=0.4991-331.6855	
Primary TB complex	3 (0.6%)	0	0	3 (1.3%)	0	3 (0.6%)
Other clinical forms of TB	492 (99.4%)	264 (100%)	260 (100%)	232 (98.7%)	12 (100%)	480 (99.4%)
Statistics	p=0.3817; OR=3.7594; 95%CI=0.1934-73.0587		p=0.1739; OR=0.1275; 95%CI=0.0066-2.4816		p=0.2684; OR=5.4914; 95%CI=0.2691-112.0748	
Miliary TB	2 (0.4%)	1 (0.4%)	0	2 (0.9%)	0	2 (0.4%)
Other clinical forms of TB	493 (99.6%)	263 (99.6%)	260 (100%)	233 (99.1%)	12 (100%)	481 (99.6%)
Statistics	p=0.9579; OR=1.0669; 95%CI=0.0963-11.8219		p=0.2680; OR=0.1793; 95%CI=0.0086-3.7535		p=0.1950; OR=7.7040; 95%CI=0.3513-168.9568	

* - statistically significant result

Table S4. Association of Beijing clusters 99-32, 94-33, 96-32 and 96-145 with clinical forms of TB among all cases (n=759)

**Diagnosis**	**99-32**	**Other Beijing**	**94-33**	**Other Beijing**	**96-32**	**Other Beijing**	**96-145**	**Other Beijing**
Disseminated TB	0	9 (1.9%)	1 (7.1%)	8 (1.7%)	1 (6.7%)	8 (1.7%)	0	9 (1.9%)
Other clinical forms of TB	11 (100%)	475 (98.1%)	13 (92.9%)	473 (98.3%)	14 (93.3%)	472 (98.3%)	11 (100%)	475 (98.1%)
Statistics	p=0.5996;<br>OR=2.1762; 95%CI=0.1193-39.6852		p=0.1675;<br>OR=4.5461; 95%CI=0.5294-39.0712		p=0.1889;<br>OR=4.2143; 95%CI=0.4930-36.0264		p=0.5996;<br>OR=2.1762; 95%CI=0.1193-39.6852	
Fibrous-cavernous TB	0	40 (8.3%)	1 (7.1%)	39 (8.1%)	1 (6.7%)	39 (8.1%)	2 (18.2%)	38 (7.9%)
Other clinical forms of TB	11 (100%)	444 (91.7%)	13 (92.9%)	442 (91.9%)	14 (93.3%)	441 (91.9%)	9 (81.8%)	446 (92.1%)
Statistics	p=0.6108;<br>OR=0.4772; 95%CI=0.0276-8.2470		p=0.8961;<br>OR=0.8718; 95%CI=0.1111-6.8412		p=0.8386;<br>OR=0.8077; 95%CI=0.1035-6.3058		p=0.2307;<br>OR=2.6082; 95%CI=0.5439-12.5065	
Infiltrative pulmonary TB	10 (90.9%)	417 (86.2%)	11 (78.6%)	416 (86.5%)	13 (86.7%)	414 (86.2%)	7 (63.6%)	420 (86.8%)
Other clinical forms of TB	1 (9.1%)	67 (13.8%)	3 (21.4%)	65 (13.5%)	2 (13.3%)	66 (13.8%)	4 (36.4%)	64 (13.2%)
Statistics	p=0.6537;<br>OR=1.6067; 95%CI=0.2024-12.7557		p=0.4021;<br>OR=0.5729; 95%CI=0.1557-2.1088		p=0.9632;<br>OR=1.0362; 95%CI=0.2296-4.6965		p=0.0392*;<br>OR=0.2667; 95%CI=0.0759-0.9367	
Focal pulmonary TB	0	4 (0.8%)	0	4 (0.8%)	0	4 (0.8%)	0	4 (0.8%)
Other clinical forms of TB	11 (100%)	480 (99.2%)	14 (100%)	477 (99.2%)	15 (100%)	476 (99.2%)	11 (100%)	480 (99.2%)
Statistics	p=0.3126;<br>OR=4.6425; 95%CI=0.2359-91.3785		p=0.3917;<br>OR=3.6590; 95%CI=0.1881-71.1887		p=0.4168;<br>OR=3.4158; 95%CI=0.1761-66.2654		p=0.3126;<br>OR=4.6425; 95%CI=0.2359-91.3785	
Tuberculous pleurisy	1 (9.1%)	6 (1.2%)	0	7 (1.5%)	0	7 (1.5%)	1 (9.1%)	6 (1.2%)
Other clinical forms of TB	10 (90.9%)	478 (98.8%)	14 (100%)	474 (98.5%)	15 (100%)	473 (98.5%)	10 (90.9%)	478 (98.8%)
Statistics	p=0.0654;<br>OR=7.9667; 95%CI=0.8759-72.4571		p=0.5993;<br>OR=2.1816; 95%CI=0.1188-40.0498		p=0.6316;<br>OR=2.0366; 95%CI=0.1113-37.2774		p=0.0654;<br>OR=7.9667; 95%CI=0.8759-72.4571	
Caseous pneumonia	0	2 (0.4%)	0	2 (0.4%)	0	2 (0.4%)	0	2 (0.4%)
Other clinical forms of TB	11 (100%)	482 (99.6%)	14 (100%)	479 (99.6%)	15 (100%)	478 (99.6%)	11 (100%)	482 (99.6%)
Statistics	p=0.1776;<br>OR=8.3913; 95%CI=0.3810-184.8271		p=0.2294;<br>OR=6.6138; 95%CI=0.3036-144.0549		p=0.2464;<br>OR=6.1742; 95%CI=0.2843-134.1077		p=0.1776;<br>OR=8.3913; 95%CI=0.3810-184.8271	
Cirrhotic TB	0	1 (0.2%)	0	1 (0.2%)	0	1 (0.2%)	0	1 (0.2%)
Other clinical forms of TB	11 (100%)	483 (99.8%)	14 (100%)	480 (99.8%)	15 (100%)	479 (99.8%)	11 (100%)	483 (99.8%)
Statistics	p=0.1117;<br>OR=14.0145; 95%CI=0.5414-362.7616		p=0.1466;<br>OR=11.0460; 95%CI=0.4313-282.8989		p=0.1581;<br>OR=10.3118; 95%CI=0.4037-263.4019		p=0.1117;<br>OR=14.0145; 95%CI=0.5414-362.7616	
Primary TB complex	0	3 (0.6%)	1 (7.1%)	2 (0.4%)	0	3 (0.6%)	1 (9.1%)	2 (0.4%)
Other clinical forms of TB	11 (100%)	481 (99.4%)	13 (92.9%)	479 (99.6%)	15 (100%)	477 (99.4%)	10 (90.9%)	482 (99.6%)
Statistics	p=0.2458;<br>OR=5.9814; 95%CI=0.2918-122.6153		p=0.0204*;<br>OR=18.4231; 95%CI=1.5694-216.2672		p=0.3340;<br>OR=4.4009; 95%CI=0.2178-88.9365		p=0.0119*;<br>OR=24.1000; 95%CI=2.0165-288.0242	
Miliary TB	0	2 (0.4%)	0	2 (0.4%)	0	2 (0.4%)	0	2 (0.4%)
Other clinical forms of TB	11 (100%)	482 (99.6%)	14 (100%)	479 (99.6%)	15 (100%)	478 (99.6%)	11 (100%)	482 (99.6%)
Statistics	p=0.1776;<br>OR=8.3913; 95%CI=0.3810-184.8271		p=0.2294;<br>OR=6.6138; 95%CI=0.3036-144.0549		p=0.2464;<br>OR=6.1742; 95%CI=0.2843-134.1077		p=0.1776;<br>OR=8.3913; 95%CI=0.3810-184.8271	

*- statistically significant result
